# Successful Treatment of Anemia With Ringed Sideroblasts Induced by Antidepressants Through Vitamin B6 Supplementation and Discontinuation of Antidepressants

**DOI:** 10.1155/crh/7046013

**Published:** 2025-07-01

**Authors:** Sanshiro Nakao, Chiaki Nakaseko, Chikako Ohwada, Keisuke Kirito, Asuka Shibamiya, Akane Tanaka, Reiko Watanabe, Naomi Shimizu

**Affiliations:** ^1^Department of Hematology, Toho University Medical Center Sakura Hospital, Chiba, Japan; ^2^Department of Hematology, International University of Health and Welfare School of Medicine, Chiba, Japan; ^3^Department of Hematology, Chiba University Hospital, Chiba, Japan

**Keywords:** antidepressants, myelodysplastic syndrome, ringed sideroblasts, sideroblastic anemia, vitamin B6 deficiency

## Abstract

Vitamin B6 (VB6) is a vital coenzyme for δ-aminolevulinic acid synthase (ALAS) in heme biosynthesis. We report a 49-year-old male with severe microcytic anemia and ringed sideroblasts initially diagnosed as myelodysplastic syndrome (MDS). VB6 deficiency, attributed to long-term amoxapine use, was identified. His anemia improved significantly with VB6 supplementation and resolved completely after discontinuing amoxapine. This case highlights the need to consider VB6 deficiency in anemia with ringed sideroblasts.

## 1. Introduction

Sideroblastic anemia is a rare type of anemia characterized by the presence of ringed sideroblasts in the bone marrow, resulting from abnormal iron accumulation in mitochondria [1–5]. It is broadly classified into hereditary and acquired forms. Acquired sideroblastic anemia includes cases associated with myelodysplastic syndrome (MDS) [6] and secondary causes such as certain medications, alcohol abuse, or other identifiable factors [7, 8]. Hereditary sideroblastic anemia, although rare, can also present in adulthood, and treatment with vitamin B6 (VB6) supplementation is effective in many cases [9–13].

VB6, also known as pyridoxine (PIN), is a vital water-soluble vitamin that plays a pivotal role in heme biosynthesis as a coenzyme for δ-aminolevulinic acid synthase (ALAS), the first enzyme in the heme production pathway. Its deficiency leads to impaired hemoglobin synthesis, resulting in microcytic, hypochromic anemia [14]. Despite its clinical significance, VB6 screening is underutilized in Japan due to a lack of insurance coverage, which may lead to low screening rates.

Here, we present the case of a 49-year-old male with severe anemia and ringed sideroblasts. He was initially diagnosed with MDS but later identified as having a VB6 deficiency caused by long-term antidepressant use. His anemia improved significantly with VB6 supplementation and resolved completely following the discontinuation of the antidepressant. This case highlights the importance of considering VB6 deficiency as a differential diagnosis in patients with sideroblastic anemia.

## 2. Case Presentation

A 49-year-old male was referred to our hospital for evaluation and treatment of transfusion-dependent severe microcytic anemia that was refractory to iron therapy. He had a medical history of depression and had been taking antidepressants (amoxapine and quetiapine fumarate) for several years, along with a history of smoking and alcohol consumption. Mild anemia had been noted for six years before presentation, with a hemoglobin level of 10.8 g/dL and a mean corpuscular volume (MCV) of 77 fL. There was no family history of anemia.

**Figure 1:**
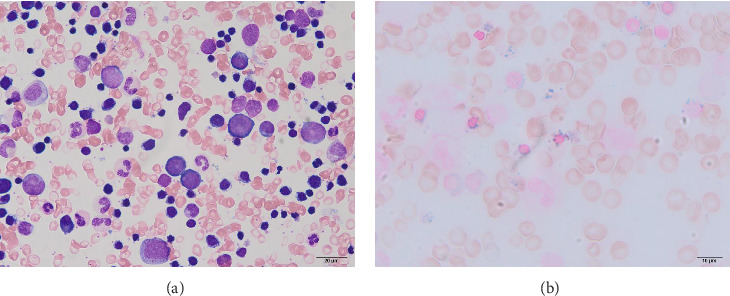
Histological findings of the bone marrow. (a) May–Giemsa staining. (b) Iron staining.

Complete blood cell counts revealed his hemoglobin level was 5.8 g/dL with a MCV value of 65 fL, platelet value of 51 × 10^4^/μL, and reticulocyte value of 0.2%. Serum iron was 261 μg/dL, total iron-binding capacity (TIBC) was 276 μg/dL, and ferritin was 727 ng/mL. Bone marrow examination showed marked erythroid hyperplasia with a myeloid/erythroid ratio of 0.4 and a myeloblast ratio of 0.4%. Erythroblasts displayed significant dysplastic features, including megaloblastic changes, irregular nuclear morphology, cytoplasmic atrophy, and vacuolization (Figure 1(a)). Most of the erythroblasts were ringed sideroblasts, with iron staining revealing Type III–V ringed sideroblasts in 98% of them (Figure 1(b)). There were no dysplastic changes in the granulocyte series. Chromosomal analysis showed a normal karyotype. Blood Wilm's tumor's 1 (WT1) mRNA was undetectable. Based on these findings, the patient was initially diagnosed with sideroblastic anemia, classified as a subtype of MDS, and required frequent red blood cell transfusions despite anabolic steroid treatment.

Considering the possibility of hereditary sideroblastic anemia, genetic testing of the causative genes of hereditary sideroblastic anemia was conducted alongside plasma VB6 measurement. This study was approved by the Institutional Review Board of the International University of Health and Welfare (Approval no. 21-Nr-051). DNA extracted from peripheral blood mononuclear cells was subjected to whole-exome sequencing to analyze causative genes, including *ALAS-2 (ALAS-E), ABCB7, GLRX5, SLC25A38, SLC25A39, SLC22A4, TMEM14C, C1orf69*, and *ISCA1* [15–18]. No mutations were identified in these genes. In addition, mutations in *SF3B1* and *SRSF2*, commonly associated with sideroblastic anemia in MDS [19], were also absent.

**Figure 2:**
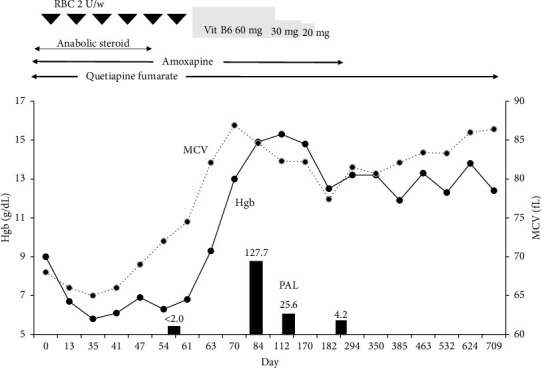
Clinical course. Vitamin B6 supplementation dramatically improved his anemia, allowing him to become free from red blood cell transfusions. However, after reducing the vitamin B6 supplementation, microcytic anemia reappeared. His anemia resolved once again following the discontinuation of amoxapine. Vit B6: vitamin B6; PAL: pyridoxal.

The VB6 study revealed undetectable levels of plasma pyridoxal (PAL), prompting the initiation of VB6 supplementation therapy (Table 1). PAL tablets (20 mg t.i.d.) were administered, leading to a dramatic improvement in anemia and normalization of MCV within 2 months (Figure 2). After gradually tapering and discontinuing VB6 supplementation, microcytic anemia recurred. The psychiatrist was contacted regarding the anemia and, based on the patient's mental status, advised discontinuation of amoxapine. After that, no changes in his mental status were observed. As shown in Figure 2, no progression of microcytic anemia was observed after the discontinuation of amoxapine, and red blood cell transfusions were no longer required.

## 3. Discussion

We report a case of adult-onset sideroblastic anemia caused by impaired VB6 absorption due to long-term amoxapine use. The patient was initially diagnosed with MDS; however, his anemia improved dramatically with VB6 supplementation, allowing him to achieve transfusion independence. Microcytic anemia reappeared after the reduction of VB6 supplementation but resolved completely upon discontinuation of amoxapine. Although PAL levels were not measured after the discontinuation of amoxapine, the improvement in hemoglobin levels following the cessation of the drug suggested that impaired absorption caused by amoxapine was likely. Since hereditary sideroblastic anemia can present in adulthood, this possibility was considered, and whole-exome sequencing of potentially causative genes was performed. No mutations were identified, making hereditary sideroblastic anemia unlikely.

VB6, also known as PIN is a water-soluble vitamin essential for numerous physiological functions. It exists in several forms, including PIN, PAL, and pyridoxamine (PAM), which are converted into the active coenzyme form, PAL 5′-phosphate, in the body. Their normal ranges are as follows: PIN ≤ 3.0 ng/mL, PAL 6.0–19.0 ng/mL, and PAM ≤ 0.6 ng/mL, respectively. Among these forms, PAL plays a crucial role in various physiological functions as a coenzyme, particularly for heme synthesis, as it supports the activity of ALAS, the first enzyme in the heme biosynthesis pathway, thereby promoting red blood cell formation.

VB6-deficient anemia is typically characterized by microcytic, hypochromic red blood cells due to impaired heme synthesis. This condition arises from a deficiency in PAL 5′-phosphate, the active form of VB6, which serves as a coenzyme for enzymes involved in hemoglobin production, including ALAS. The deficiency leads to inadequate heme production and may cause iron accumulation in mitochondria, resulting in sideroblastic anemia in severe cases.

Hematological findings in VB6-deficient anemia often reveal low hemoglobin levels and low MCV, with ringed sideroblasts in the bone marrow being a hallmark feature in advanced stages. Clinically, patients may experience fatigue, weakness, and pallor, which are common symptoms of anemia. These symptoms may overlap with other manifestations of VB6 deficiency, such as peripheral neuropathy, irritability, or depression.

VB6 deficiency occurs due to various causes, including insufficient dietary intake, malabsorption, chronic alcoholism, certain medications, or genetic disorders affecting VB6 metabolism. It impairs enzymatic activity, particularly in pathways involving PAL 5′-phosphate, the active form of VB6. Secondary acquired sideroblastic anemia due to impaired absorption of VB6 has been reported in patients treated with drugs such as levodopa/carbidopa for Parkinson's disease [20]. Other medications associated with VB6 deficiency include antituberculosis drugs, antibiotics, and anticancer agents [21]. In this case, there have been no prior reports of VB6 deficiency anemia caused by antidepressants, and amoxapine is thought to be the causative drug, as the anemia improved after discontinuation of this medication. Alcohol is another potential contributor to VB6 deficiency, as it can interfere with VB6 metabolism [22]. Regarding the effect of alcohol intake on VB6 metabolism, it has been reported that blood concentrations of plasma PAL-5′-phosphate, the active coenzyme form of VB6, are significantly low in alcohol drinkers. However, in this case, the patient stopped drinking alcohol after his initial hospital visit, making it unlikely to have played a role. Importantly, VB6-deficient anemia typically responds well to VB6 supplementation, leading to rapid improvement in hemoglobin levels and normalization of red blood cell indices.

Sideroblastic anemia with VB6 deficiency is an important differential diagnosis in sideroblastic anemia associated with MDS. Recently, the efficacy of luspatercept has been reported in the treatment of anemia in low-risk MDS, particularly in cases with ringed sideroblasts [23, 24]. Therefore, the possibility of VB6-deficient anemia should be investigated before considering luspatercept for sideroblastic anemia associated with MDS.

In adult patients with sideroblastic anemia, adult-onset hereditary sideroblastic anemia is also an important differential diagnosis. In hereditary sideroblastic anemia, treatment with VB6 is the first-line therapy, and significant improvement in anemia is observed in many cases. The patient showed dramatic improvement with VB6 supplementation, prompting consideration of hereditary sideroblastic anemia as a possibility. Genetic analysis of potentially causative genes such as *ALAS-2* was performed, but all results were negative, ruling out hereditary sideroblastic anemia. [18]. Furthermore, we also analyzed the mutation of *SF3B1* and *SRSF2*; their mutations were frequently observed in MDS, but no mutations were identified in these genes.

In conclusion, it is essential to consider the possibility of VB6 deficiency in addition to MDS for patients presenting with anemia and ringed sideroblasts. Early recognition and timely treatment of VB6 deficiency are critical for preventing anemia-related complications and improving overall health outcomes.

## Figures and Tables

**Table 1:** Laboratory data at the first visit.

Hematology			Biochemistry			Others			Bone marrow findings	
WBC	6600	/μL	CRP	0.31	mg/dL	Vitamin B6			NCC (× 10^4^/μL)	22.9
Seg	77.5	%	TP	6.3	g/dL	PAM	≤ 0.2	ng/mL	Mgk (/μL)	60
Ly	15.0	%	ALB	4.4	g/dL	PAL	≤ 2.0	ng/mL	M/E ratio	0.4
Mono	5.5	%	T-BIL	0.6	mg/dL	PIN	≤ 3.0	ng/mL		
Eos	0.0	%	AST	15	IU/L				Chromosome	
Baso	0.0	%	ALT	10	IU/L	FER	727	ng/mL	46, XY	20/20
RBC	290	× 10^4^/μL	LDH	119	U/L	Fe	261	μg/dL		
Hgb	5.8	g/dL	ALP	68	U/L	UIBC	15	μg/dL	Fe staining	
Ht	18.8	%	G-GT	41	IU/L	TIBC	276	μg/dL	0	0%
MCV	65.0	fl	BUN	9.9	mg/dL	EPO	234	mIU/mL	I	0%
MCH	20.4	pg	CRE	0.67	mg/dL	Vitamin B12	381	pg/mL	II	1%
MCHC	31.9	%	UA	5.0	mg/dL	Folic acid	7.9	ng/mL	III	12%
PLT	51.0	× 10^4^/μL	Na	140	mEq/dL				IV	54%
Ret	0.2	%	K	4.3	mEq/dL	WT1	< 50	copy/μg	V	32%
			Cl	103	mEq/dL	JAK2 V617F	(−)			
			Ca	9.7	mg/dL					
			BS	113	mg/dL					

*Note:* PAM, pyridoxamine; PAL, pyridoxal; PIN, pyridoxine; WT1, Wilms' tumor 1.

## Data Availability

The data supporting this study's findings are available from the corresponding author upon reasonable request. However, the data are not publicly available due to privacy or ethical restrictions.
